# Evaluation of techniques for performing cellular isolation and preservation during microgravity conditions

**DOI:** 10.1038/npjmgrav.2016.25

**Published:** 2016-07-14

**Authors:** Lindsay F Rizzardi, Hawley Kunz, Kathleen Rubins, Alexander Chouker, Heather Quiriarte, Clarence Sams, Brian E Crucian, Andrew P Feinberg

**Affiliations:** 1 Center for Epigenetics, Johns Hopkins University School of Medicine, Baltimore, MD, USA; 2 Science, Technology and Engineering Group, Wyle, Houston, TX, USA; 3 Astronaut Office, NASA Johnson Space Center, Houston, TX, USA; 4 Department of Anesthesiology, Hospital of the Ludwig-Maximilians-University, Munich, Germany; 5 JES Tech, Houston, TX, USA; 6 Space and Clinical Operations Division, NASA Johnson Space Center, Houston, TX, USA; 7 Biomedical Research and Environmental Sciences Division, NASA Johnson Space Center, Houston, TX, USA; 8 Departments of Medicine, Biomedical Engineering, and Mental Health, Johns Hopkins University Schools of Medicine, Engineering, and Public Health, Baltimore, MD, USA

## Abstract

Genomic and epigenomic studies require the precise transfer of microliter volumes among different types of tubes in order to purify DNA, RNA, or protein from biological samples and subsequently perform analyses of DNA methylation, RNA expression, and chromatin modifications on a genome-wide scale. Epigenomic and transcriptional analyses of human blood cells, for example, require separation of purified cell types to avoid confounding contributions of altered cellular proportions, and long-term preservation of these cells requires their isolation and transfer into appropriate freezing media. There are currently no protocols for these cellular isolation procedures on the International Space Station (ISS). Currently human blood samples are either frozen as mixed cell populations (within the CPT collection tubes) with poor yield of viable cells required for cell-type isolations, or returned under ambient conditions, which requires timing with Soyuz missions. Here we evaluate the feasibility of translating terrestrial cell purification techniques to the ISS. Our evaluations were performed in microgravity conditions during parabolic atmospheric flight. The pipetting of open liquids in microgravity was evaluated using analog-blood fluids and several types of pipette hardware. The best-performing pipettors were used to evaluate the pipetting steps required for peripheral blood mononuclear cell (PBMC) isolation following terrestrial density-gradient centrifugation. Evaluation of actual blood products was performed for both the overlay of diluted blood, and the transfer of isolated PBMCs. We also validated magnetic purification of cells. We found that positive-displacement pipettors avoided air bubbles, and the tips allowed the strong surface tension of water, glycerol, and blood to maintain a patent meniscus and withstand robust pipetting in microgravity. These procedures will greatly increase the breadth of research that can be performed on board the ISS, and allow improvised experimentation by astronauts on extraterrestrial missions.

## Introduction

The ability to collect human biosamples on board the International Space Station (ISS) is fairly limited. With rare exception, only frozen urine, saliva, or blood plasma are routinely collected and stored. Recently, the return of ambient blood samples has occurred allowing expanded analyses, but this is constrained to only two samplings per 6-month ISS mission owing to availability of the returning Soyuz vehicles. There would be a scientific benefit for ISS to expand the frequency of flight sampling and to isolate purified cells from blood or tissue while on board the ISS, including peripheral blood mononuclear cells (PBMCs) or positively isolated cell populations (e.g. CD4^+^, CD8^+^, or CD19^+^ cells). This capability would facilitate a host of cellular and omics analyses for which segregation of distinct cell populations has become increasingly important. It is generally perceived that techniques for isolating cells, including density-gradient centrifugation (DGC) with Ficoll solution or magnetic separation of cells, are gravity dependent. This perception is based on the assumption that liquid human biosamples would be difficult to manipulate in microgravity and could represent a biological hazard if uncontained, or that sensitive density-dependent steps such as overlaying of fluids or removal of cellular bands would be compromised without gravity. We postulated that such steps could be performed safely in microgravity using standard pipetting techniques. Further, the development of septum-based tubes to facilitate DGC has the potential to render these standard techniques easier in microgravity. These tubes are constructed with a barrier between the Ficoll solution and blood layers instead of relying on a sensitive overlay, yet with pores in the insert that allow cellular translocation upon centrifugation. After centrifugation, the barrier protects the isolated PBMCs from contamination by red blood cells or granulocytes, which would be advantageous on-orbit. We evaluated the fluid-handling steps required for DGC, including the loading of Ficoll solution, the overlay of blood, and the removal and preparation for cryopreservation of isolated cells, but not centrifugation itself in microgravity conditions. Multiple pipetting techniques were evaluated, as were three types (and two sizes) of DGC tube hardware. All evaluations were performed on the NASA C-9 parabolic flight laboratory aircraft. These evaluations demonstrated that the pipetting of open fluids is relatively simple and easily controlled and that all fluid transfer steps associated with DGC can be replicated in microgravity. Observation of actual purified blood products indicated that the layers created during DGC remain stable in each type of tube evaluated during microgravity. Further, the overlay pipetting of actual diluted blood and the removal of isolated PBMCs was easily accomplished.

## Results

### Testing multiple methods for liquid transfer

**Figure 1:**
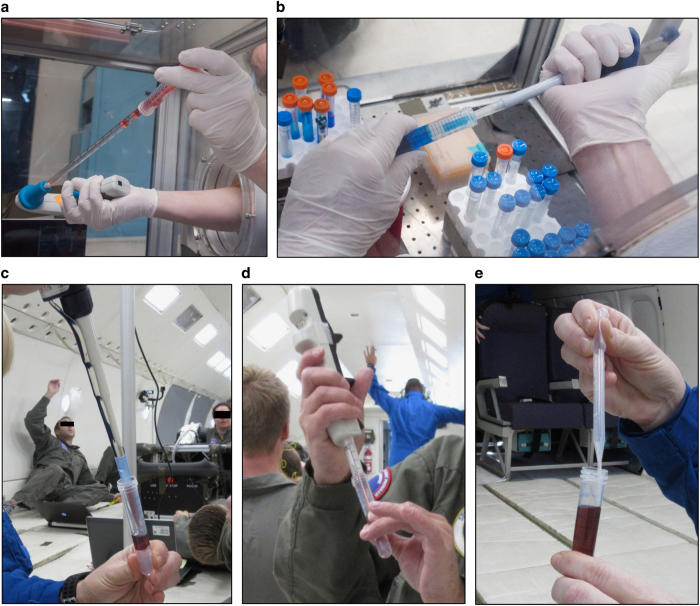
Evaluation of different pipettes for liquid transfer. (**a**) Serological pipette (10 ml) used to transfer dyed water into a 15 ml conical tube. (**b**) Positive-displacement pipette (1 ml) used to transfer dyed water into a 15 ml conical tube. (**c**) Standard air-displacement laboratory pipette (1 ml) used to transfer dyed water into a 12 ml LeucoSep tube. (**d**) Positive-displacement repeater pipette (5 ml) used to transfer dyed water into a 12 ml LeucoSep tube. (**e**) Plastic bulb transfer pipette used to transfer dyed water into a 12 ml LeucoSep tube.

**Figure 2:**
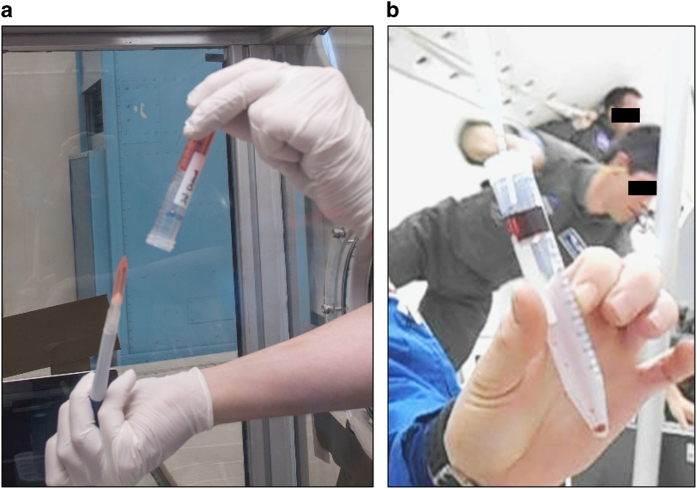
Liquid behavior in microgravity. (**a**) Liquid in the tubes does not spontaneously float out of the tubes, no matter how the tube is oriented. (**b**) Fluids can be easily affixed in place by pipetting anywhere in the tube.

We tested five different methods for liquid transfer using several types of pipettors (Figure 1) in microgravity based on several assumptions. The first assumption was that liquids in microgravity would be set in motion by surface tension effects and consequently would not necessarily remain in their tubes when opened during pipetting. To counteract this assumed outcome, we utilized a completely closed system known as a cannula transfer (Supplementary Figure 1) commonly used in chemical synthesis protocols.^1^ In order to transfer liquids from one tube to another, the tubes have septum lids that can be punctured by long needles connected to each other via Tygon tubing. Injection of air into the liquid-containing tube increases the pressure in the tube causing the liquid to move up through the needle and tubing into the empty tube. During our initial parabolic evaluations, we discovered that our assumptions were incorrect and that the liquid in the tubes would not spontaneously migrate (Figure 2). The spontaneous movement of fluids, without gravity restraint, generally depends on the wetting characteristics of the fluid, the composition and diameter of the tube itself, and how full the tube is (dependent on the first two conditions). We concluded that the surface tension acting on the liquid in the tubes was sufficient to counteract the reduction in gravity. In addition, the capillary effects were insufficient to cause fluid movement. Nonetheless, we proceeded to test the cannula transfer system. We found that once set up, the transition during the parabola from 1.8*g* to microgravity was sufficient to initiate the transfer of liquid through the tubing. Although this unassisted transfer would not occur in constant microgravity (as on board the ISS), the fluid reached a surface tension equilibrium that disrupted our control of the subsequent liquid transfer. We had difficulty forcing the liquid through the tubing after the initial fluid transfer and would occasionally get backflush into the original tube while performing parabolas. Given the complex set up and lack of controlled liquid transfer we eliminated the cannula system as a viable method for transfer.

We next tested pipetting pure water with several common laboratory pipettors. For reference, pipettors are the entities held in the hand to control the pressure in the pipette (alters speed of fluid uptake and dispensation); pipettes are the entities into which fluid is drawn and dispensed except in the case of micropipettors where this is referred to as the pipette tip. Our assumption in this case was that as water was drawn up into the pipette tip the reduced gravity would cause (1) the liquid to continue to flow up the inside of the tip and into the pipettor itself (thus contaminating the pipettor) and as a result of this would (2) prevent accurate liquid measurement. The first method we tested utilized plastic serological pipettes (5 and 10 ml capacity) and a battery-operated pipette-aid. Our assumptions were unfounded and we had no issues with uncontrolled water flowing up the inside of the serological pipette (Figure 1a). However, we found that as we inserted the serological pipette into the tube of water we displaced a volume of the liquid that proceeded to migrate up along the inside of the tube. As the pipette was inserted, the wetting, capillary, and surface tension characteristics of the liquid were likely responsible for adherence to the side of the tube and migration along the tube as the pipette tip was inserted further. The migrated liquid would stay at the top of the tube until jostled by the experimenter, at which time the droplet would float away. During the removal of the liquid-containing pipette, a small quantity of liquid would sometimes adhere to the pipette itself, and a droplet would break off and float away. This significant finding illustrates the importance of the speed of pipette removal as well as accounting for wetting effects. Although not directly measured, we observed that slow removal of the pipette tip reduced droplet adherence to the pipette tip and reduced the likelihood of jostling by the experimenter. Aside from this issue, we were able to pipette pure water successfully with this method. The difficulty was preventing the introduction of a bubble while doing so, as the migrating liquids altered the liquid level in the tube requiring adjustments to where the pipette tip was placed during the drawing up of the liquid. Bubble formation and release characteristics vary significantly in microgravity compared to terrestrial conditions as has been shown previously.^2–4^ For these reasons we eliminated this method with the caveat that a larger diameter tube (such that the surface tension is not substantially altered and which allows for less bulk fluid displacement when the serological pipette is inserted) could allow successful use of the serological pipette for transfer.

Next, we tested a plastic (specifically, low-density polyethylene polymer) bulb transfer pipette (1 ml gradations; 3.1 ml bulb draw). Although the least accurate of the pipettes tested, it performed adequately for pipetting water, glycerol, blood, and Ficoll solution analogs, and isolated PBMCs (Figure 1e). A problem arose when liquid was drawn into the bulb portion of the pipettor from which it could not be dislodged (as also occurs at 1*g* and is not attributable to microgravity conditions); however, as long as liquid was drawn up slowly and with control, the transfer pipette was shown to be a viable liquid transfer option. Owing to the smaller width of the tip, there were no fluid displacement or surface tension problems as seen with the serological pipettes. Altogether, these results suggest that any tips utilized in microgravity should have a diameter no more than half that of the tubes that will be used (Table 1).

The final laboratory pipettors tested were a standard air-displacement laboratory pipettor (1 ml capacity; Figure 1c) and two types of positive-displacement pipettors (PDP): an Eppendorf positive-displacement repeater pipettor (Figure 1d) and Gilson PDPs (capacities ranging from 10 μl–1 ml; Figure 1b and Supplementary Video 1) that each use a piston within the tip to allow accurate pipetting while preventing contamination and carry-over. We chose to test this style of pipettor in addition to a traditional air-displacement pipettor to prevent contamination of the pipettor itself in the event of uncontrolled liquid flow. However, our concerns were unfounded as the standard air-displacement pipettor performed just as well as the PDPs in this regard. These were by far the most successful pipettors tested. We easily and accurately transferred water, glycerol (10 and 100%), and PBMCs using these laboratory pipettes (Figure 1). Precision was assessed visually, for example, by dispensing 1 ml of liquid into a conical tube and noting that the liquid came up to the 1 ml gradation indicated on the tube. Further, in pipetting 1 ml up and down, we noted that the same volume was repeatedly dispensed and retrieved. The only issue we had was again due to the width of the 1 ml Gilson PDP (Middleton, WI, USA) tip relative to the tube diameter when used with the cell preparation tubes (CPT; CPT Vacutainer blood collection tubes containing sodium citrate and a polyester gel plug over a Ficoll-Hypaque density solution layer; cat. no.: 362760, BD Biosciences, San Jose, CA, USA), which have a much smaller diameter than the 15 ml conical tubes (Table 1). As long as the very end of the pipette tip (smallest diameter) was kept in contact with the liquid, this issue was minimized. This issue was not observed with the repeat pipettor and lower capacity Gilson PDP tips owing to the smaller tip diameters. Although it was much less of an issue with the 1 ml PDP than with the serological pipettor tips, in future, we recommend the use of tips with a tip:tube diameter ratio ≤0.5 for pipetting in microgravity such as those used with the positive-displacement repeater pipette or Gilson PDPs.

### Demonstration of pipetting solutions for density separations

**Figure 3:**
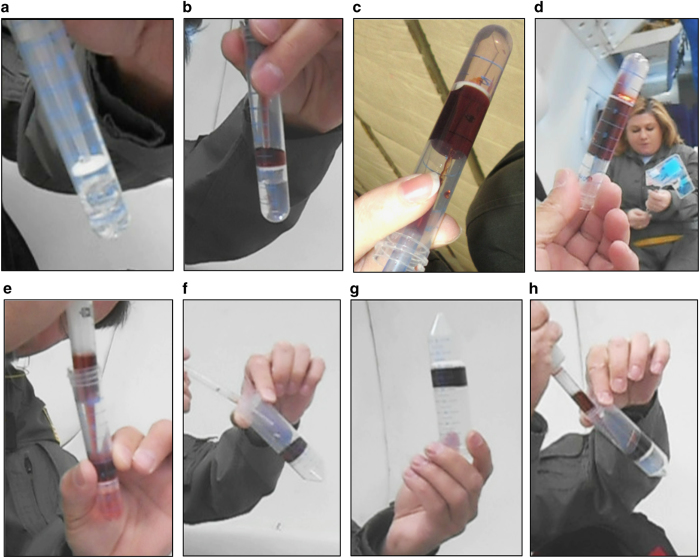
Video capture images documenting evaluation of the steps required to set up a density-gradient centrifugation in microgravity conditions. Note that all centrifugation steps were performed terrestrially immediately prior to flight. Analog fluids were used: blood analog was dyed water while Ficoll solution analog was 10% glycerol in PBS. (**a**) Loading of a LeucoSep 12 ml tube with Ficoll solution analog. (**b**) Overlay of blood analog. (**c**) Demonstration that liquids pipetted from ~2 cm away from the barrier (but touching the side wall) are still captured in the appropriate position for the overlay. (**d**) Evidence that mild manipulations (such as slight tilting of the tube back and forth) did not disturb the integrity of the overlay step. (**e**) Extraction of the overlaid blood analog. (**f**) Overlay of a LeucoSep 50 ml tube with blood analog. (**g**) Image demonstrating that when placed in their proper position, the fluids generally remained in place even for a large-bore 50 ml tube, facilitating operations in microgravity. (**h**) Extraction step for the 50 ml LeucoSep tube. PBS, phosphate-buffered saline.

The steps required for proper execution of a density-based cell separation (loading of Ficoll solution, overlay of sample, and removal of layers) were evaluated for three types of separation tubes: LeucoSep 12 ml tube (cat. no.: 163289, Greiner Bio-One, Kremsmünster, Austria), LeucoSep 50 ml tube (cat. no.: 227289, Greiner Bio-One) and a SepMate 15 ml tube (cat. no.: 15415, Stemcell Technologies, Vancouver, BC, Canada). Each of these tubes contain a barrier that separates the Ficoll solution from the diluted blood, eliminating the need for a careful overlay. The barrier in the LeucoSep tubes is a porous polyethylene, whereas the insert in the SepMate tube has several relatively larger pores through which cellular translocation occurs. The 50 ml LeucoSep tube is the only one tested that lacks a central hole in the barrier for adding density-gradient media and is loaded via centrifugation of Ficoll solution. This fluid loading step could not be performed on board the aircraft and was performed terrestrially prior to parabolic flight. For those tubes containing the central hole (LeucoSep 12 ml and SepMate 15 ml), the loading of Ficoll solution in microgravity was tested by loading water and 10% glycerol (‘analog Ficoll’) below the barrier. It was generally found that the push of fluid through the pore into the confined space was easily performed. Whereas in gravity, a pipetted liquid will ‘fall’ and displace air, in microgravity the introduced liquid intermingled with air, resulting in bubbles (Figure 3a). However, we found that the necessary volume of analog Ficoll solution could be properly positioned at the bottom of the tube below the insert using a mild centrifugal force generated in-flight by holding the tube at its top and gently swinging it outward at arm’s length to create a resultant force toward the bottom of the tube.

Next, the overlay pipetting step was evaluated for the 12 ml LeucoSep and 15 ml SepMate DGC apparatus using dyed water (analog-blood liquid) transferred via three pipetting techniques. Note that terrestrially, the overlay is facilitated by gravity, with the introduced fluids being ‘held’ in place over the loaded Ficoll solution, but even in microgravity, the analog blood was held in place over the analog Ficoll solution. Fluid was easily aspirated, and then repeatedly pipetted precisely into position creating an acceptable overlay (Figure 3b). We found the overlay was not dependent on carefully ‘placing’ the fluids with the pipette; even pipetting the liquids from ~2 cm away from the interface without contact with the interior surface of the tube allowed capture and collection of the liquid for overlay in the appropriate position (Figure 3c). Further, manipulations of the tube in microgravity did not disturb the overlaid fluids, as evidenced by Figure 3d. Extraction of the overlaid fluids, essentially another aspiration step, which for evaluation purposes was performed without a centrifugation step, was then easily performed (Figure 3e). As the behavior of the open liquids was easily controlled in both of the tubes, the protocol was repeated for the larger diameter 50 ml LeucoSep tubes. As with the smaller tubes, the overlay of analog-blood was easily performed (Figure 3f). We found it remarkable that even in the larger bore tube, fluids continued to remain in place despite manipulation (Figure 3g). Extraction of the overlaid fluids from the 50 ml tubes was also easily performed in microgravity (Figure 3h). (Note: all liquid handling during these evaluations was performed using a standard 3.1 ml bulb transfer pipette, an Eppendorf positive-displacement repeater pipette, and a standard 1000 μl air-displacement pipette, each of which performed well.) Video at https://youtu.be/5YSeYkCxp4Y provides video support for the results associated with Figure 3.

### Observations of separated blood products in microgravity

**Figure 4:**
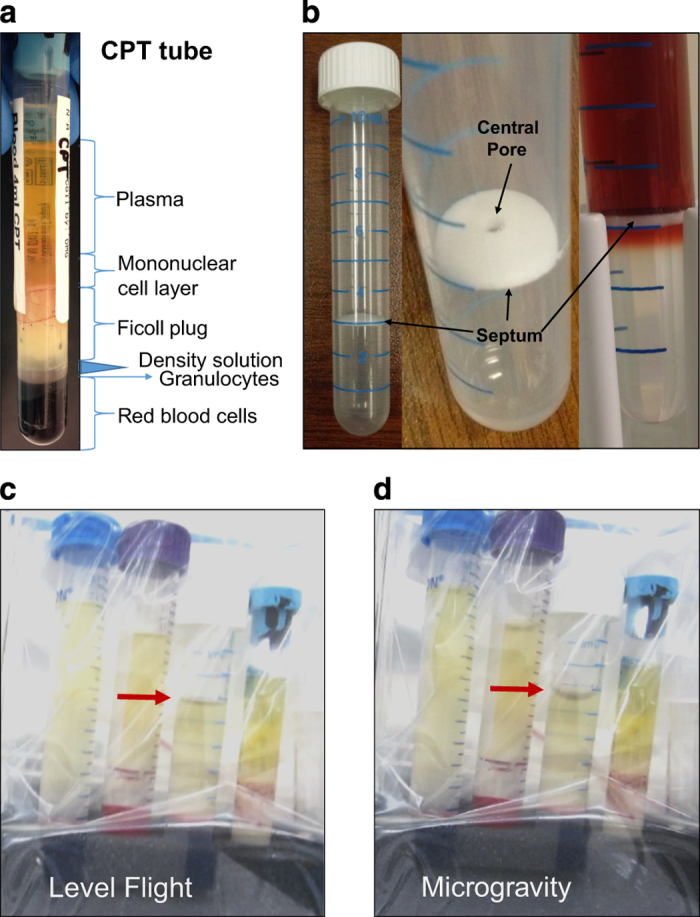
Observation of the behavior of actual blood products (processed in a BD CPT Vacutainer tube using terrestrial density-gradient centrifugation) during 1*g* level flight versus microgravity conditions. (**a**) Example of processed blood sample in a BD CPT Vacutainer tube. (**b**) Greiner Laboratories LeucoSep tube designed to facilitate density-gradient centrifugation, possibly enabling such techniques to be performed in microgravity conditions. The LeucoSep tube possesses a physical porous barrier, or insert, which separates the blood (or analog fluid) above from the Ficoll solution (or analog) fluid below. A central pore (indicated) allows loading of the Ficoll solution below the barrier, and allows cellular displacement between the layers during centrifugation. Terrestrially, gravity continues to ‘assist’ in maintaining physical separation between the layered components, even after centrifugation. In the LeucoSep tube, the barrier protects the integrity of the separated components after centrifugation, a feature highly desirable for microgravity conditions. (**c**, **d**) Blood products processed and centrifuged prior to flight via standard Ficoll DGC in, from left to right: a 15 ml conical tube; a 15 ml SepMate tube; a 12 ml LeucoSep tube; and via the BD CPT Vacutainer tube. Representative images are shown for both level flight and microgravity parabolic flight. Tubes were flown triple-contained and video was recorded during several parabolas to ensure blood sample behavior was similar to that observed for the analog fluids used in the pipetting evaluation (Figure 3). Note the meniscus of the upper plasma layer (red arrow for the LeucoSep 12 ml tubes) is altered during microgravity conditions, but in all the tubes the fluids maintained their positions and were never displaced within the tube. CPT, cell preparation tubes.

The blood samples were processed through the centrifugation step terrestrially in normal gravity prior to flight for four types of DGC hardware, but the resulting PBMC ‘band’ (Figure 4a), was not extracted. These processed tubes were then observed during microgravity conditions to evaluate the physical interactions between the blood products and the various tube hardware. The fluid layers were undisturbed, and the tubes were maintained in a vertical position (under triple containment). The four tubes were a LeucoSep 12 ml tube, a SepMate 15 ml tube, a BD CPT Vacutainer, and a standard 15 ml conical tube. The LeucoSep 12 ml tube possesses a physical porous barrier, or insert, with a central pore for loading Ficoll solution below the insert while keeping the blood (or analog fluid) above the Ficoll solution layer (Figure 4b). The upper layer of the processed samples, consisting of plasma and platelets, and containing the PBMC band, remained in place during three consecutive parabolic maneuvers which consisted of several altered gravity states (level flight (1*g*), 1.8*g*, and microgravity; Figure 4c,d). The flat interface during level flight appears slightly curved owing to the optical effect of the cylindrical tube and the angle of photography. Video at https://youtu.be/xArZFaBL_tk provides support for Figure 4. The only deviation was a slight elevation of the meniscus between the plasma and air space within the LeucoSep tube indicated by the red arrows in Figure 4c,d. After exiting the microgravity period, the meniscus returned to its previous level flight status. These slight alterations did not result in disruption of the PBMC band.

### Overlay pipetting of diluted blood and recovery of PBMCs in microgravity

**Figure 5:**
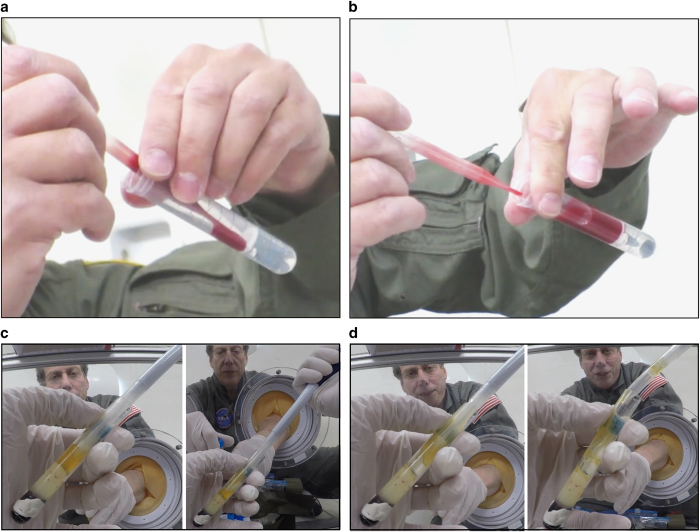
Preparation of a whole-blood sample for density-gradient centrifugation using a LeucoSep 12 ml tube. The LeucoSep tube was preloaded with Ficoll solution and a whole-blood sample diluted 1:1 with PBS was carried aboard in a separate tube. (**a**) Using a 3.1 ml bulb transfer pipette, the blood was overlaid onto the porous device barrier in microgravity, effectively yielding a true microgravity preparation of a density-gradient separation. (**b**) Resulting microgravity preparation for a density-gradient separation. (**c**,**d**) To test the extraction step with actual blood products, PBMCs were collected during microgravity using a positive-displacement pipette from CPT Vacutainer tubes prepared terrestrially prior to parabolic flight. (**c**) Slow and careful pipetting allows for successful aspiration of PBMCs while rapid and careless pipetting (**d**) makes it impossible to extract precise volumes of PBMCs. CPT, cell preparation tubes; PBMCs, peripheral blood mononuclear cell.

To confirm the above findings with actual blood samples, we evaluated overlay pipetting of actual whole EDTA blood diluted 1:1 with phosphate-buffered saline (PBS) over Ficoll solution. The LeucoSep 12 ml tube was used for this evaluation and was loaded with Ficoll solution terrestrially prior to the flight. Pipetting was performed using the 3.1 ml bulb transfer pipette. Blood was aspirated into the transfer pipette, and subsequently overlaid onto the porous insert of the 12 ml LeucoSep tube. Interestingly, we found that there were mild alterations in the behavior of the diluted whole blood. Rather than being easily affixed in place, the blood was slightly ‘sticky’ with a mild affinity for the sidewalls of the tube as evidenced in Figure 5a,b. Video at https://youtu.be/xArZFaBL_tk provides support for Figure 5. Nevertheless, it was still feasible, with slightly more care than required for the analog-blood fluid (dyed water), to pipette the blood into place and successfully create the overlay (Figure 5b).

To evaluate the removal of PBMCs in microgravity from CPT Vacutainer tubes, we collected the blood samples and centrifuged the tubes at Johnson Space Center prior to parabolic flight just as current ISS on board protocols dictate. The total time from centrifugation to observation in microgravity was ~2 h due to logistics prior to flight. As the PDPs performed the best, we used the 1 ml capacity PDP to withdraw the PBMCs from the CPT Vacutainer tube (Figure 5c,d). PBMCs (~3 ml) were collected from the CPT Vacutainer tube by resuspending them in human plasma (top layer in CPT tube; Figure 4a) and transferring them into a 15 ml conical tube containing 333 μl of 100% glycerol for a final glycerol concentration of 10%. Each pipetting step was performed during the microgravity portions of a single parabolic flight excursion with intervening 1–1.8*g* states between (and was repeated on a subsequent flight day). We confirmed in our own lab prior to flight that this concentration of glycerol was sufficient to allow freezing and thawing of viable PBMCs. We observed that re-suspending the cells in the plasma as well as mixing with the glycerol can be done without generating bubbles; however, the pipette tip must be kept towards the top of the liquid and mixing via pipetting up and down must be done very slowly to accomplish this (Figure 5c). The CPT Vacutainer tubes are the smallest diameter tubes we tested and if liquid was dispensed too rapidly or the pipette tip displaced too much liquid, bubbles would form or liquid would crawl up the sides of the CPT Vacutainer tube making it very difficult to impossible to precisely aspirate the PBMCs (Figure 5d and Table 1). The PBMC/glycerol mixture was distributed into 3–4 cryovials during the microgravity portions of parabolic flight and stored on ice (brought on board the aircraft in a Styrofoam container) for the remainder of the flight. After landing, they were transferred to dry ice for transport to Johnson Space Center and stored at −80 °C.

### Quality analysis of isolated PBMCs

Microgravity-collected PBMCs (referred to as ‘ZeroG frozen’) were shipped on dry ice to Johns Hopkins University for further processing. As a control, we isolated and collected PBMCs in the lab from a different donor via the same protocol used during parabolic flight. One sample was collected and frozen just as for the microgravity sample (referred to as ‘Hopkins frozen’). The other sample (referred to as ‘Hopkins ambient’) was centrifuged in the BD CPT Vacutainer tube and stored at 4 °C overnight to mimic the current protocols used for the NASA Twin Study. Cells were thawed (when necessary) and total cell number and viability were measured both before and after isolating the CD4^+^ and CD8^+^ fractions via magnetic separation with Miltenyi MicroBeads (Miltenyi, Auburn, CA, USA; Table 2). DNA was extracted from each cell type, quantified, and bisulfite converted. Sequencing libraries were generated and checked for quality before destroying the samples. The average fragment size was 441 bp. There were no major differences in the quality or quantity of libraries generated from terrestrially collected PBMCs versus PBMCs collected in microgravity (Table 2). Therefore, human PBMCs collected on board the ISS and stored as described here would be suitable for downstream –omic analyses.

### Evaluation of magnetic separation in microgravity

**Figure 6:**
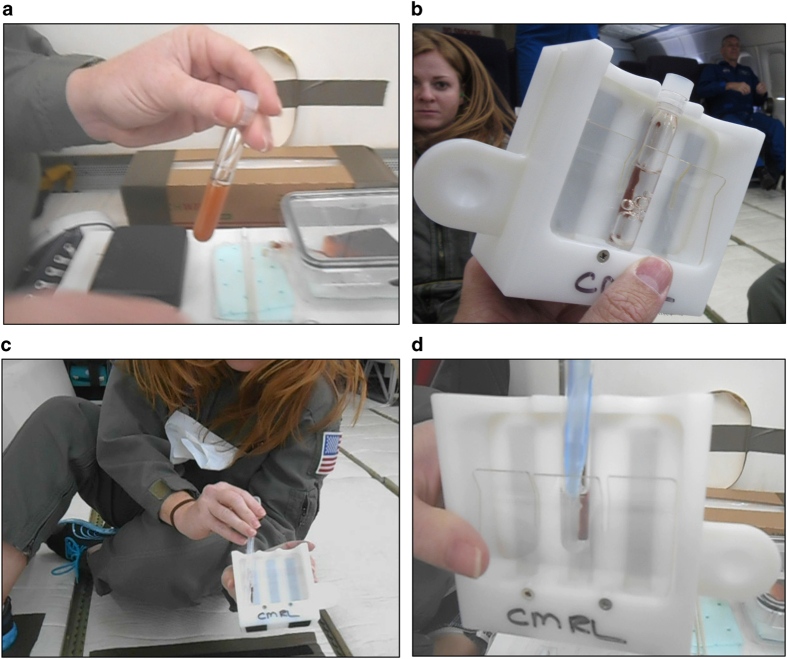
Evaluation of magnetic separation using Dynabeads. (**a**) Dynabeads uniformly suspended in PBS. (**b**) Magnetic separation of beads using a Dynabead separation magnet. (**c**,**d**) Removal of the liquid phase without disturbing the magnetic beads using a transfer pipette (**c**) and a standard air-displacement pipette (**d**).

The basic pipetting steps associated with magnetic cell separation were evaluated during microgravity. Dynabeads (Thermofisher, Waltham, MA, USA) suspended in PBS in a 12×75 polystyrene tube were carried on board the parabolic aircraft (Figure 6a). During microgravity, the magnetic separation was performed by placing the tube in a Dynabead separation magnet (Figure 6b). Separation occurred within 30 s of microgravity, confirming the assumption that this step would be gravity-independent, as even terrestrially the magnetic force is dominant over gravity, allowing separation in a typical laboratory setting. It was observed that bubbles would permeate the liquid and not ‘rise’ and thus be eliminated from the system (Figure 6b). However, the presence of bubbles did not seem to inhibit the separation and a proper isolation of the magnetic beads on the tube side wall. Extraction of the liquid phase, required for subsequent cell purification, was easily performed with either a bulb transfer pipette (Figure 6c) or a standard 1,000-μl air-displacement laboratory pipette (Figure 6d). Video at https://youtu.be/UZINFN0gERQ depicts the steps associated with magnetic separation performed in microgravity conditions.

## Discussion

Although common perception is that handling of open human biological liquids would be difficult or unsafe in microgravity, we found that all pipetting steps related to DGC were easily performed in microgravity using analog fluids as well as human blood products (Figure 1). We previously observed that glass and plastic blood collection tubes fill differently in microgravity. In plastic tubes blood tends to fill ‘top to bottom’ in any orientation, whereas blood has a greater affinity for glass vacutainers. In glass tubes, blood tends to rapidly ‘coat’ the interior surface, resulting in a hollow ‘core’ of air until the tube completely fills (authors’ unpublished observations from parabolic flight and spaceflight on board ISS). This observation in part contributed to our assumption that liquid handling (particularly of actual blood) would be quite difficult during microgravity conditions. However, we found that processed blood products and Ficoll solution behaved similarly to their analogs (water and glycerol) and could easily and accurately be manipulated via pipetting (Figure 5 compared with Figure 2).

We tested a variety of pipetting mechanisms and found that the most important factor for success was the ratio of diameters between the pipette/tip and the liquid-containing tube. The air-displacement, positive-displacement, and transfer pipettes performed the best for each liquid and tube type tested as they had the smallest tip diameters compared with the larger serological pipettes (Table 1). At no point during microgravity did liquid spontaneously flow out of the tubes as we initially expected (Figure 2). The most difficult aspect of liquid handling proved to be preventing the introduction of bubbles into the liquids during transfer or mixing. Once bubbles were introduced, there was no way to remove them or to pipette around them during microgravity. This finding underscores the absolute requirement for very careful and slow pipetting to ensure bubbles are never introduced.

After determining that the PDPs and standard laboratory air-displacement pipettes are the most accurate and easy-to-use methods of liquid transfer, we have a few recommendations to further simplify liquid transfer using this method. The first is to utilize a 5 ml capacity pipettor (e.g., ErgoOne 5 ml pipettor; cat. no.: 7150–5000, USA Scientific, Ocala, FL, USA), which would prevent multiple pipetting steps for larger volumes (such as in the CPT Vacutainer tubes), decreasing the opportunities for introducing bubbles. Care needs to be taken to insert only the end of the tip (2 mm diameter) as the diameter increases to 11 mm towards the middle of the pipette tip. Ideally, smaller diameter tips could be manufactured for this pipettor to prevent the fluid displacement and surface tension issues we demonstrated in microgravity. For smaller volumes, the currently available PDPs and air-displacement pipettors (ranging in capacity from 10 μl to 1 ml) perform well. Second, a ‘space-ready’ gripper box for the pipette tips would allow the box to remain open while holding the tips in place (Supplementary Video 2). A tube holder that allows the user to snap various sizes of tubes in place (perhaps using a snap clamp such as those used for vortex mixers) would also be helpful.

This study has found that all steps needed for DGC and cell collection can be completed in microgravity conditions. The deployment of a swinging bucket centrifuge to ISS (currently anticipated in 2017) will be the final necessary hardware to allow purified mononuclear cells to be easily collected on board ISS. The steps required for cell collection (following terrestrial DGC; Figure 5) and magnetic bead separation of cells (Figure 6; both pipetting and magnetic ‘pull’) were demonstrated during this parabolic flight evaluation. Coupling DGC with magnetic separation would allow the collection of purified types of peripheral leukocytes on board ISS. This capability would greatly enhance on board human biosample collection for various types of –omics analyses, with the potential to complement existing biosample repository collections.

## Materials and methods

### Biological and analog samples

The microgravity evaluation was primarily performed using analog fluids. Dyed water represented blood, and 10% glycerol in 1× PBS (Sigma, St. Louis, MO, USA) represented Ficoll solution. 100% glycerol was also tested to determine how very viscous fluids would perform in microgravity. EDTA blood was collected from a single healthy test subject. Prior to flight, the EDTA sample was diluted 1:1 with isotonic 1× PBS. Ficoll solution (Sigma) was flown unaltered. Blood from two additional healthy test subjects was collected into BD CPT Vacutainer tubes. The NASA laboratory maintains an IRB approval for blood sample collection related to assay development and instrument validation, including microgravity testing, which applied to this activity.

### Evaluation hardware

Three types of hardware which support density-gradient separation were evaluated during microgravity conditions: LeucoSep 12 ml, LeucoSep 50 ml, and SepMate 15 ml tubes. Each of these tubes possesses a porous barrier which separates the Ficoll solution from the sample (prior to centrifugation) and isolates the PBMC and plasma layers from the red blood cells and granulocytes layers (after centrifugation). Five types of pipettes were used: a standard bulb transfer pipette (1 ml gradations; 3.1 ml bulb draw), serological pipettes with battery-powered pipette-aid (5 and 10 ml), an Eppendorf positive-displacement repeater pipette, a series of Gilson positive-displacement pipettes (10–1,000 μl), and a standard 1,000 μl (blue tip) pipette (Table 1). We also tested a cannula transfer system that consisted of 15  ml conical tubes with septum lids that can be punctured by long needles (18G, 3.5 inch Tuohy Epidural needles) connected to each other via luer-locks and Tygon tubing. One 15 ml tube contained dyed water while the other was empty. Liquid transfer was initiated by injecting air into the water-filled tube via a standard 10 cc syringe and 25G needle. We also tested performance of magnetic Dynabeads (Thermofisher, Waltham, MA, USA) along with the Dynabead magnet (Thermofisher) with blood and Ficoll solution analogs.

### Microgravity conditions

NASA can perform actual near-microgravity evaluation of hardware on Earth by using parabolic flight aircraft. A NASA C-9 jet aircraft flies in a parabolic arc, where an approximately 30 s period of microgravity is generated at the top of the parabola, and a corresponding period of 1.8 *g* is generated at the pull-up from the dive. On a flight, 40 parabolas are flown in a repeated sequence, resulting in 40 ~30-s microgravity opportunities. The ‘microgravity’ period of 30 s during atmospheric parabolic flight may have very minor fluctuations of either positive or negative *G* forces. These fluctuations may be introduced by atmospheric phenomenon as the pilot maintains the parabolic maneuver, and may cause any released object to drift either slightly upward or downward. However, the 30-s condition is generally considered sufficient for a microgravity hardware evaluation. The evaluation described herein occurred on four consecutive flight days, and all steps were performed within ~30 s of microgravity. Flight logs available upon request. Gravity states were constantly measured throughout the flight and flight logs are available upon request.

### Protocol for transferring PBMCs from CPT Vacutainer tube in microgravity

On the ground, blood was drawn directly into a CPT Vacutainer tube, which was inverted 10 times, incubated at room temperature for 15 min, and centrifuged at 1,800*g* for 20 min at room temperature to separate the PBMC layer (Figure 4a). The CPT Vacutainer tube was then taken onto the aircraft for the microgravity parabolas (~2-h delay). In accordance with ISS safety requirements, the CPT tube was triply contained when taken on board the aircraft and immediately transferred into the glove box for all subsequent experiments. All the sample handling was done in microgravity (with the on-board gravity indicator at zero) with pauses at each gravity transition (from 1.8*g* to microgravity). While working inside a glove box, a 1 ml PDP was used to transfer the topmost 1 ml of plasma into a 15 ml conical tube. After the next *g*-transition, we mixed the remaining plasma carefully by pipetting to re-suspend the cell layer while in microgravity. During microgravity periods, we sequentially transferred 1 ml of this cell suspension into a 15 ml conical tube until <1 ml remained. Owing to the low volume and width of the pipette tip we had to use a transfer pipette for the remainder; this would have been unnecessary if we had used the repeater PDP, which has a much narrower tip (Table 1). We estimated the volume from the incremental measurements displayed on the tube. We added 100% glycerol to a final concentration of 10% using an appropriately sized PDP. We pipetted up and down carefully to thoroughly mix, transferred 900 μl into each of three cryovials and placed on ice (all pipetting occurred during the microgravity portions of parabolic flight). Once they had been mixed, we did not observe any separation of the glycerol and cell mixture during *g*-transitions. Upon landing, the tubes were transferred to dry ice and then to an insulated container to freeze at −80 °C.

### Cell type isolation at Johns Hopkins using Miltenyi magnetic beads

Data in Table 2 were obtained as follows. Frozen aliquots of PBMCs (‘Hopkins frozen’ and microgravity samples—‘ZeroG frozen’; described in Results section ‘Quality analysis of PBMCs) were thawed rapidly in a 37 °C water bath, combined in a 15 ml conical tube (if from the same sample), and washed twice with 10 ml of 1× PBS, pH 7.5. PBMCs isolated from the ‘Hopkins ambient’ sample were also washed twice in 10 ml 1× PBS. Cells were collected by centrifugation at 300*g* for 10 min at 4 °C and resuspended in 5 ml of cold Cell Suspension Buffer (CSB; 1× PBS containing 2 mmol/l EDTA, 0.5% bovine serum albumin). Total cell count and viability were measured using the Countess Automated Cell Counter (Invitrogen, Carlsbad, CA, USA). Cells were spun again at 300*g* for 10 min at 4 °C and resuspended in 80 μl CSB. To isolate CD4^+^ cells, 20 μl of CD4^+^ magnetic beads (cat. no.: 13004510, Miltenyi) was added, mixed by pipetting, and incubated 15 min on ice in the dark without agitation. Following the incubation, 5 ml of cold CSB was added and the samples were centrifuged at 300 *g* for 10 min at 4 °C. To capture the CD4^+^ cells, the pellet was resuspended in 1 ml CSB and passed through the MS columns attached to a magnet stand (Miltenyi) as per the manufacturer’s instructions. The flow through was saved and used for the isolation of CD8^+^ cells following the same protocol, but using the CD8^+^ magnetic beads (cat. no.: 130045201, Miltenyi). Once the CD4^+^ cells were captured on the column, a 500-μl CSB wash was performed. To elute the CD4^+^ cells into a 1.5 ml Eppendorf tube, the column was removed from the magnet, 1 ml of CSB was added, and the included plunger was used to expel the CD4^+^ cells. Total cell and viability counts for the CD4^+^, CD8^+^, and the remaining unlabeled cell fraction were performed using the Countess Automated Cell Counter (Invitrogen). The cells were pelleted by centrifugation at 300*g* for 10 min at 4 °C and DNA was isolated using the MasterPure DNA Purification kit (Epicentre, San Diego, CA, USA). Total DNA was quantified using the Qubit dsDNA BR Assay kit (Invitrogen). The DNA was stored at −20 °C until use.

### Bisulfite conversion and library prep

Whole-genome bisulfite sequencing single-indexed libraries were generated using NEBNext Ultra DNA library Prep kit for Illumina (New England BioLabs, Ipswich, MA, USA) according to the manufacturer’s instructions with modifications. Five hundred nanograms of genomic DNA was quantified by Qubit dsDNA BR assay (Invitrogen) and fragmented by Covaris S2 sonicator to an average insert size of 350 bp. Size selection was performed using AMPure XP beads and insert sizes of 300–400 bp were isolated. Samples were bisulfite converted after size selection using the EZ DNA Methylation-Gold Kit (Zymo, Irvine, CA, USA) following the manufacturer’s instructions. Amplification was performed after the bisulfite conversion using Kapa Hifi Uracil+ (Kapa Biosystems, Boston, USA) polymerase using the following cycling conditions: 98 °C 45 s followed by 8 cycles of: 98 °C 15 s, 65 °C 30 s, 72 °C 30 s; and a 1-min incubation at 72 °C.

Final libraries were run on 2100 Bioanalyzer (Agilent, Santa Clare, CA, USA) High-Sensitivity DNA assay, samples were also analyzed on Bioanalyzer after shearing and size selection for quality control purposes. Libraries were quantified by qPCR using the Library Quantification Kit for Illumina sequencing platforms (KAPA Biosystems, Boston, MA, USA), using the 7900HT Real Time PCR System (Applied Biosystems, Waltham, MA, USA).

## Figures and Tables

**Table 1:** Tube and pipette tip characteristics

*Supplier*	*Cat. no.*	*Tip*	*Plastic composition*	*Outer diameter (mm)*	*Ratio to 15 ml conical (15-mm inner diameter)*	*Ratio to CPT (10.5-mm inner diameter)*
USA Scientific	1122–1830	1 ml XL filter tip	Polypropylene	5.5	0.367	0.524
USA Scientific	1075–0110	5 ml serological pipette	Polystyrene	8	0.533	0.762
USA Scientific	1071–0810	10 ml serological pipette	Polystyrene	9.5	0.633	0.905
USA Scientific	1111–2821	1 ml standard pipette tip	Polypropylene	7	0.467	0.667
Sigma	Z135070	3.1 ml transfer pipette	Polyethylene	5	0.333	0.476
USA Scientific	4751–0500	5 ml repeat pipette tip	Polypropylene (outer); Polyethylene (piston)	3–5	0.2–0.33	0.28–0.47
Gilson	CP1000	1 ml PDP tip	Polypropylene (outer); polyacepal (piston)	7.4	0.493	0.705
Gilson	CP250	50–250-μl PDP tip	Polypropylene (outer); polyacepal (piston)	4	0.267	0.381

Abbreviations: CPT, cell preparation tubes; PDP, positive-displacement pipettors.

All diameters were measured at the middle of pipette tip.

**Table 2:** CD4^+^ and CD8^+^ cell isolation results

*Sample*	*Cell type*	*Total cells*	*Viability*	*Viable cells*	*Total isolated DNA (ng)*
Hopkins Ambient	All Cells	9×10^6^	56%	5.22×10^6^	NA
	CD4^+^	1.5×10^6^	63%	945,000	702
	CD8^+^	1.1×10^6^	72%	800,000	678
	CD4^−^/CD8^−^	5.2×10^6^	60%	3.2×10^6^	1,890
					
Hopkins Frozen	All Cells	5.7×10^6^	50%	2.85×10^6^	NA
	CD4^+^	850,000	63%	535,000	570
	CD8^+^	420,000	73%	310,000	660
	CD4^−^/CD8^−^	2.4×10^6^	33%	780,000	330
					
ZeroG Frozen	All Cells	5.7×10^6^	45%	2.56×10^6^	NA
	CD4^+^	840,000	59%	495,600	882
	CD8^+^	990,000	45%	450,000	672
	CD4^−^/CD8^−^	1.76×10^6^	38%	660,000	1,038

Abbreviation: NA, not applicable.
