# Supplementary material for: Evaluation of techniques for performing cellular isolation and preservation during microgravity conditions
Source: NPJ Microgravity. 2016 Jul 14;2:16025–. doi: 10.1038/npjmgrav.2016.25 (PMC5515526; doi:10.1038/npjmgrav.2016.25)
Supplement: Supplemental Figure 1 [file npjmgrav201625-s1.pdf]

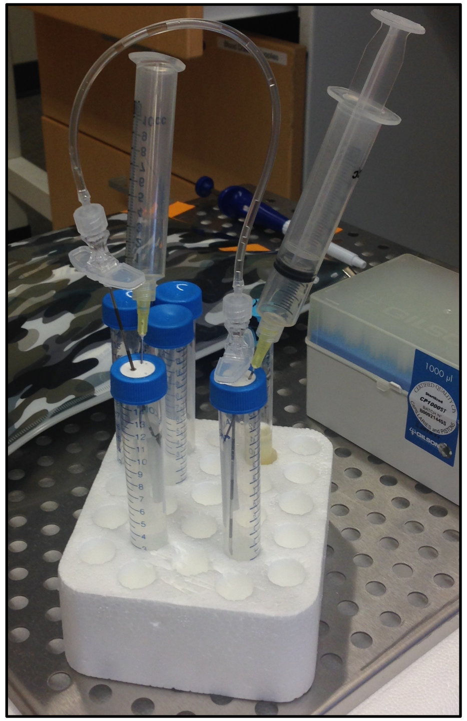

Supplemental Figure 1. Terrestrial set up of cannula transfer. Tygon tubing attached to two needles connects two 15 ml conical tubes with and without liquid. A syringe is used to inject air into the liquid containing tube to force the liquid through the tubing into the empty tube. A syringe without a plunger is used to vent the displaced air from the empty tube as liquid enters.
